# Structuralizing Culture: Multicultural Neoliberalism, Migration, and Mental Health in Santiago, Chile

**DOI:** 10.1007/s11013-024-09858-4

**Published:** 2024-05-24

**Authors:** Gabriel Abarca-Brown

**Affiliations:** 1 Center for Culture and the Mind (CULTMIND), Department of English, Germanic and Romance Studies (ENGEROM), University of Copenhagen, Emil Holms Kanal 6, 2300 Copenhagen, Denmark; 2 Universidad Diego Portales, Santiago, Chile

**Keywords:** Migration, Multicultural neoliberalism, Structure-based approach, Intersectionality, Chile

## Abstract

The arrival of Afro-descendant migrants, mainly from Haiti and the Dominican Republic, has led to the emergence of new discourses on migration, multiculturalism, and mental health in health services in Chile since 2010. In this article, I explore how mental health institutions, experts, and practitioners have taken a cultural turn in working with migrant communities in this new multicultural scenario. Based on a multisited ethnography conducted over 14 months in a neighbourhood of northern Santiago, I focus on the Migrant Program—a primary health care initiative implemented since 2013. I argue that health practitioners have tended to redefine cultural approaches in structural terms focusing mainly on class aspects such poverty, social stratification, and socioeconomic inequalities. I affirm that this structural-based approach finds its historical roots in a political and ideological context that provided the conditions for the development of community psychiatry experiences during the 1960s and 1970s, as well as in multicultural and gender policies promoted by the state since the 1990s. This case reveals how health institutions and practitioners have recently engaged in debates on migration and intersectionality from a structural approach in Chile.

## Introduction

The arrival of Afro-descendant migrants, mainly from Haiti and the Dominican Republic, has led to the emergence of new discourses on migration, multiculturalism, and mental health in health services in Chile since 2010. For health institutions and practitioners, Afro-descendant migration has triggered, unlike the migratory groups that arrived in the country after the end of the civic-military dictatorship (1973–1989)—as was the case in the Peruvian and Bolivian migrations, among others—a series of frictions, conflicts, and challenges. Specifically, mental health and sexual and reproductive health practitioners have faced practices typical of Haitian-Creole medicine and Haitian Vodou, while experts and patients have alerted and complained about racist attitudes in clinical and community spaces. Both the Ministry of Health and the local Health Department in charge of primary healthcare have tried to respond to practitioners’ demands. They have promoted several training sessions on “migration and human rights,” “cultural competency,” and “Haitian culture,” aimed at strengthening what they called—as I will develop later—“intercultural health.” However, this cultural shift has triggered various reactions from health teams. While some practitioners have valued adopting a culturally informed approach to health, others have questioned its success.

In this article, I explore how mental health institutions, experts, and practitioners have taken a cultural turn in working with migrant communities in this new multicultural scenario. I focus on the “Migrant Program”—a primary healthcare initiative implemented by the Health Department of a northern Santiago neighborhood since 2013. Integrating both central guidelines and local actions on migration and health, the Migrant Program sought to improve access to health services, eradicate racism, and promote culturally relevant interventions in teams.

I argue that although health institutions and experts have encouraged a cultural turn to meet migrants’ health needs, health practitioners have tended to redefine cultural approaches in structural terms, highlighting the role of the categories of “class” and “gender,” mainly. In other words, rather than adopting a cultural-based approach, practitioners approached migrants’ afflictions and health trajectories from a structural-based perspective focusing primarily on class aspects such as poverty, social stratification, and socioeconomic inequalities. I affirm that this approach finds its historical roots in a political and ideological context that provided the conditions for the development of community psychiatry experiences—specifically during the reformist governments between 1965 and 1973^1^—as well as from multicultural and gender policies promoted by the state since the 1990s.

In this article, I do not limit the discussion to public health and global mental health debates regarding the relevance and scope of cultural competency and structural competency models. Cultural competency is an educational agenda developed by medical schools in the US in the 1990s that seeks to improve the ability of healthcare practitioners to understand and engage with individuals from diverse cultural backgrounds effectively. It involves gaining knowledge and awareness of different cultural norms, beliefs, values, and practices and applying that understanding to deliver healthcare services (Office of Minority Health, 2001). However, a few years later, some anthropologists criticized this approach, arguing that it reproduces stereotypes (Taylor, 2003) and shapes ethnic taxonomies (Kleinman & Benson, 2006). In this scenario, another US-based group of anthropologists and health practitioners advocated for considering structural factors in health (Holmes, 2011; Metzl & Hansen, 2014). Thus, the structural competency model gradually became relevant in health practitioners’ training. This model provides a frame on how broader social, economic, and political conditions (e.g., socioeconomic inequalities, racism, gender violence, among others) shape afflictions and well-being and produce inequalities in health (Metzl & Hansen, 2014). It also encourages practitioners’ training based on fundamental principles, such as understanding patients’ experiences in the context of structural factors; addressing structural factors at an institutional level (e.g., schools, social services, and the police); and community collaboration and connectivity, and structural humility (Hansen et al., 2018)^2^

Rather than limiting the scope of the discussion to the relevance of competency models employed by institutions and healthcare practitioners when working in transcultural contexts, this article adopts a broader historical and anthropological lens. It seeks to shed light on the transmission and perpetuation of health practices in Chile throughout recent decades, beginning in the 1960s. Moreover, it delves into the practitioners' understanding of “the social” (Adams et al., 2019) and their utilization of the categories of “culture” and “structure” (Jenks, 2010). By doing so, this article aligns with existing scholarly contributions that emphasize the structural dimensions of health approaches in Latin American countries (Harvey et al., 2022; Harvey & Piñones-Rivera, 2023; Ortega & Müller, 2022; Ortega & Wenceslau, 2020).

This article is based on a research project that sought to examine how new discourses and practices related to migration, multiculturalism, and mental health have emerged in neoliberal postdictatorship Chile (1990–2019). Specifically, I explored how the introduction of health reforms and the global mental health agenda have impacted and shaped the subjectivity and everyday life of Haitian and Dominican migrants. For this, I carried out a multisited ethnography (Marcus, 1995) over 14 months in a neighborhood of northern Santiago during 2018–2019. I conducted observation sessions in a Family Health Center (CESFAM, in Spanish), in social organizations and churches, and in other migrants' everyday life spaces. Additionally, I conducted 45 interviews with experts, health practitioners, Haitian and Dominican migrants and their families, evangelical pastors, and Vodou healers.^3^

This article is divided into four sections. First, I describe the Migrant Program's main actions between 2013 and 2019. The program encouraged a cultural turn, implementing activities oriented to neighbors and health practitioners. However, the program’s practitioners gradually criticized this turn due to the lack of resources for reaching their goals. From this, I highlight how the program can be framed within what some Chilean anthropologists have called “multicultural neoliberalism” (Bolados, 2010; Bolados García, 2012), a radical version of the “neoliberal multiculturalism” described in other Latin American countries (Hale, 2005; Richards, 2016). This form of multiculturalism is not simply a reduced version of multiculturalism (Hale, 2005), but rather an expansion of neoliberalism into previously overlooked sociocultural realms such as “intercultural health” (Bolados García, 2012). Subsequently, I describe how the program developed training sessions on “cultural competency” and “Haitian culture” in 2018 and 2019. I show that although health teams valued knowledge provided by experts in those sessions, many practitioners advocated for a “more complex form of cultural competency.” With this, they criticized how the cultural competency model “essentializes” the relationships between culture and health and “reproduces stereotypes.” At the same time, those practitioners revitalized a “way of doing mental health” in which the categories of “class” (e.g., poverty, social stratification, and socioeconomic inequalities) and “gender” were crucial. Here, I highlight how, unlike structural-based approaches described mainly by US–Canada-based researchers (Kirmayer et al., 2018; Metzl & Hansen, 2014), practitioners' approach in Chile tended to downplay the significance of other categories such as race, sexuality, and ability. Third, I show that this mental health approach results from practices anchored in community psychiatry experiences during the reformist governments (1965–1973) and the adoption of multicultural and gender policies since the 1990s. Finally, I discuss the relevance of adopting a historical and anthropological perspective in public health and global mental health debates on cultural competency and structural competency.

## The Migrant Program: Introducing a Cultural Turn in the Health Field

Yolanda^4^ and Miriam—social workers from the Migrant Program—reviewed some documents for the health intervention (“el operativo de salud”) with a group of family doctors and nurses at the CESFAM lobby that Saturday morning. The initiative sought to screen (“pesquisar”) cases of tuberculosis in a sector of the neighborhood near the center, as well as to determine the living conditions of the neighbors and inform them about the different health benefits. For one of the CESFAM family doctors, the screening was urgent due to the detection of a case of tuberculosis a couple of weeks earlier. For their part, Yolanda and Miriam considered that the intervention was an excellent opportunity to determine the levels of “poverty” and “overcrowding” among the neighbors of that sector, which was characterized by the significant number of Latin American and Caribbean migrants. A few days before the “operativo,” Yolanda told me:

The screening is a great opportunity because we can work with biomedical practitioners and with the community in the territory in health prevention and promotion. For me, this is something unique… practitioners who come voluntarily on a Saturday (...) Perhaps, some time ago, they [practitioners] only thought about the relationship between a specific disease and poverty. For example, tuberculosis with living conditions (…) But now due to the [Migrant] Program, they are forced to ask themselves, for example, how do Haitian migrants understand tuberculosis? Or how could migrants understand what a preventive test means?

The local Health Department launched the Migrant Program in 2013 as a response to a series of frictions, conflicts, and challenges in public health centers triggered mainly by the arrival of Latin American and Caribbean migrants to the neighborhood during the last two decades. Health practitioners encountered various issues including access barriers, discrimination complaints, and challenges in delivering mental health and sexual and reproductive health services (Abarca-Brown, 2023, 2024). The department established this initiative as a “transversal program” within primary healthcare. Its objective was to address multiple objectives simultaneously, such as improving health service accessibility, combating racism, and integrating migration and ethnic–cultural aspects into health programs for children, adolescents, and adults. To achieve this, the program sought to familiarize CESFAM teams, comprising family doctors, dentists, nurses, midwives, psychologists, social workers, and paramedics, with these topics.

The Ministry of Health funded the Migrant Program for three years after 2015. Considering different initiatives developed in separate cities around the country, the Ministry sought to systematize “good practices,” aiming to create a “Health Policy for International Migrants.” With this, the Migrant Program increased its budget three times. The new resources were allocated mainly^5^ to the hiring of Haitian “cultural facilitators” and organizing training sessions on “migration and human rights,” “cultural competency,” and “Haitian culture.” University professors, experts, and NGO workers lectured in these sessions. In the context of an interview, Yolanda said:

The facilitators helped practitioners by translating from Creole to Spanish and sometimes mediating cultural issues (…). I think facilitators have greatly helped in the clinical space (…). But I think the training sessions have been important because they have more scope. To be honest, we have not followed any specific training program. It has been something more improvised. We have been guided more by the needs we have detected each year. For example, we started with training sessions in migration and human rights, then in cultural competency and Haitian culture. Here, we have seen, for example, the role of Vodou, of traditional healers (…). We have tried to reach all the practitioners of the neighborhood.

In general terms, the Health Department and practitioners valued the program’s actions. However, when I started my ethnographic work in 2018, Yolanda and Miriam were critical of practitioners’ views on “intercultural health.” They said practitioners’ perspective on “intercultural issues” was “very narrow.” Practitioners mostly used the term “intercultural” to refer to the mere encounter with migrant and indigenous communities in clinical and community settings. For Yolanda and Miriam, this use was far from Latin American traditions in intercultural health (Harvey et al., 2022; Menéndez, 2016), which they were familiarized with due to their experience working in community interventions in other countries of the region. Besides, Yolanda and Miriam were critical of the program’s scope. They pointed out that “the state does not support these multicultural initiatives enough” and “the program's budget does not guarantee its continuity over time.” Miriam critically noted:

We need more facilitators. We have only two facilitators for all the centers (...). We don't have a budget to pay the presenters for the training sessions. Some come here through volunteering, but you can't always ask them to come for free (...). Without funding, the program limits its action to creating informative material in Creole or paying some facilitators (...). This focus on linguistic aspects is not new. It has happened with Mapuche communities (…). For example, the actions limit themselves to putting up informative posters inside the health centers in another language. For example, in Mapudungun.^6^ Nothing else. As Yolanda always says, "we are in diapers" in intercultural health (...). The work should focus on the intercultural relationship between the health system, practitioners, and patients.

Yolanda and Miriam's critiques resonated with what, as I mentioned earlier, some researchers have called “multicultural neoliberalism” in Chile (Bolados, 2010; Bolados García, 2012). This version of multiculturalism differs from the “neoliberal multiculturalism” described in other countries of the region (Boccara, 2007; Boccara & Bolados, 2010; Hale, 2005; Richards, 2016). Neoliberal multiculturalism operates mainly at the level of the recognition of Otherness in ethnic terms, but that does not integrate a political and economic redistribution that addresses structural change. While other Latin American countries developed multicultural policies in the 1970s and 1980s, the Chilean state committed to recognizing indigenous communities after the end of the civil-military dictatorship in 1989 (Boccara, 2007; Boccara & Bolados, 2010). Unlike neighboring countries such as Peru and Bolivia, the Chilean reformist governments and social movements of the 1960s and 1970s did not adopt “mestizo nationalism” as a reference (Richards, 2016). In other words, they did not integrate the idea of a mestizo nation as a source of collective pride and resistance to neocolonial domination. On the contrary, Chilean reformist movements conceived indigenous issues mainly from a class perspective (e.g., Mapuche as a poor subject), and subsequently, the civic-military dictatorship (1973–1989) ignored these issues.

The Chilean multicultural approach has sought to “protect” and “activate” specific vulnerable ethnic groups (e.g., Mapuche, Aymara) through minimal subsidies and limited social services benefits rather than guaranteed social rights (Navarrete Saavedra, 2022). Multicultural policies have encouraged these communities to “empowerment” themselves and find better social and economic “opportunities” and relegated any indigenous demands related to “autonomy,” “redistribution of land,” and “self-determination” (Navarrete Saavedra, 2022). Within this frame, the extension of neoliberalism towards sociocultural fields such as “intercultural health” is materialized in different health programs and actions specially oriented to indigenous populations since the 1990s. For example, the Chilean state implemented—supported economically by the Inter-American Development Bank—the Origins Program (“Programa Orígenes”). This program aimed at developing and improving the quality of life for the Mapuche, Aymara, and Atacameño people. It was the first program to convene social actors and leaders interested in making the indigenous health system visible. The program supported the carrying on of “ancestral and intercultural health meetings,” where the so-called “demands for recognition of indigenous medicine” were systematized (Bolados García, 2012). Different researchers have argued that this program conceptualized indigenous communities as “corporative groups” (Bolados García, 2012) and overlooked power relationships in the health field (Rivera et al., 2017).

Multicultural neoliberalism took form in the scarcity of resources and the lack of technical support for the continuity of the Migrant Program initiatives. Yolanda and Miriam constantly expressed their frustration with the Ministry of Health for ending funding. However, despite the uncertainty surrounding the program’s future actions, Yolanda and Miriam lived with resignation and acceptance of that situation. Both agreed that the program should be aimed at maintaining cultural facilitators and the “constant familiarization” of practitioners with the cultural competency model. In turn, they conveyed fear that the program limited itself to mainly the creation of informative material in Creole. Yolanda said, “this is public health in Latin American countries. Sometimes we have money, sometimes we don’t. But we always push forward with determination.”

## Structuralizing Culture

Training sessions became less frequent because of budget cuts in 2018. Since then, the program could not organize more than five training sessions in each CESFAM per year. Due to this new scenario, Yolanda and Miriam focused their efforts on events on “cultural competency” and “Haitian culture,” aiming to achieve “constant familiarization” among the health teams. To achieve this, the program invited university professors and researchers, NGO workers, and experts who voluntarily agreed to give a talk on these issues. Like Yolanda and Miriam, those experts mainly highlighted how Haitian-Creole medicine and Vodou challenged mental health practitioners’ backgrounds and everyday work. Haitian-Creole medicine is a set of knowledge, practices, and values related to health and illness comprised of practices from biomedicine, herbal medicine, and Vodou (Damus & Vonarx, 2019).^7^ Vodou is not only a religion but also a health system that interacts with biomedicine and other religions (Damus & Vonarx, 2019; WHO, 2010). Vodou, as a worldview, places the subject within a universe of ancestors, spirits, and the natural world (“lwa”). For this reason, both the representation of the “person,” “health,” and “disease,” as well as their classification are composed of natural and supernatural categories (Vonarx, 2012).

Those experts, who were generally Haitian with training in public health and medical anthropology, focused on areas that practitioners considered critical, such as mental health and sexual and reproductive health (Abarca-Brown, 2023, 2024). They provided many examples based on clinical cases in Haiti and in different host countries of the Haitian diaspora, such as the US and Canada. Through these cases, they tried to explain why patients did not attend or abandoned psychological consultations, the various symptomatic manifestations of different conditions, and the contents of hallucinations and delusions. In an interview, one of these experts said:

The first thing that health workers must understand is the role of Vodou as a health system (...). Then, they must understand that the centrality of the Vodou worldview in Haiti is not the same as being a Vodou practitioner (...). And after this, the workers will understand the relationship that Haitian patients have with the CESFAM, psychologists and different psychopathological manifestations.

In general, CESFAM’s practitioners valued these training sessions, particularly those focused on Haitian-Creole medicine and Vodou. Some of them appreciated the possibility of “taking a little distance from the biomedical model.” In a conversation regarding the sessions with one of the CESFAM’s psychologists, she referred:

It’s been a very short time that we've known about the importance of Vodou in Haitian culture (...) Now we understand a little more about the forms of hallucinations or trances... or the beliefs and rituals of pregnant women attended by midwives in the Chile Crece^8^ (…). After these sessions you feel that you have more tools for working with Haitian migrants. But we need more of these trainings, and these [training sessions] have to reach all the teams.

In the same line, one of the family doctors at the center said in an interview:

For me, the training sessions have been very useful (...). When you realize that in Haiti, there is no official mental health system as we understand it, you realize that the doctor, psychiatrist, and psychologist are not valid figures for the patient. Why should they tell things about their private life to someone they do not know? Or for example, depression tends to have somatic symptoms in the Haitian population, so they don’t go to the psychologist, they go to the doctor.

To a large extent, the psychologist’s and family doctor’s words represented the general position of CESFAM’s practitioners about the relevance of adopting a cultural perspective in their work. The co-production of knowledge between the invited experts and the practitioners provided dynamism to the clinical and community encounters between the teams and the Haitian migrants. Indeed, some practitioners said that the workshops gave them “more tools” for their work and that they could “understand Haitians better.”

Nonetheless, some practitioners who participated in these sessions on “cultural competency” and “Haitian culture” gradually adopted a skeptical position regarding the Migrant Program approach since the second half of 2018. This skepticism rested on two pillars. First, there is criticism of the public health system’s precariousness, particularly mental health services. CESFAM’s psychologists and social workers whom I worked with usually pointed out that mental health is the “abandoned area of Chilean public health.” For some of them, due to the scarcity of resources, health policies and primary healthcare actions prioritized what they called “morbidity” and “chronic patients.” That is, interventions oriented to acute diseases and high-burden health conditions such as “hypertension” and “diabetes,” respectively. They also argued that these actions led to “biomedical teams”—comprised mainly of family doctors and nurses—receiving more resources from the Ministry of Health and the local Health Department. While I was talking with a CESFAM group of psychologists regarding the training sessions, one of them said:

We see 12 or 14 patients a day, you have 30 minutes to attend to a patient and fill out the [clinical] record (...) with a Haitian patient time flies because the facilitator has to translate (...). I love my job [“pega”]. I would love to work well in an intercultural way (...) or have clinical meetings to talk about intercultural things. However, honestly, this is exhausting [“el cuero no da”] (…). You want to be critical, intercultural, and blah blah, but, at the end, you are the most biomedical of the biomedical practitioners [laughing].

Precariousness seemed to mark a material limit regarding the scope of the usual interventions in mental health and the adoption and assimilation of new practices based on a cultural approach. The psychologist’s words show, as other ethnographies have similarly described on in northern Santiago (Han, 2012), how some practitioners' actions are anchored in a particular political and ethical commitment rather than policies and programs with adequate resources.

These commitments—what they usually called “vocación”—played a crucial role when mental health practitioners faced health system precarity. Psychologist and social workers who identified themselves as more involved in “community psychology” and “critical perspectives” faced the lack of resources by encouraging community interventions as a cost-effective strategy (e.g., greater number of users per intervention). For them, practitioners had to prioritize actions that they regularly carried out at the primary healthcare level. For instance, they promoted actions such as home visits and community-oriented interventions in neighborhood councils, schools, and sports clubs, among others. Based on their experience in the Migrant Program training sessions, some of these practitioners emphasized the relevance of working with migrants—specially Haitians—in their everyday spaces such as Evangelical churches. However, as I will describe in the following section, other practitioners contested this community approach based on a how health system and goals have been framed in a economic rationalite during the last years.

The second pillar took form in what these practitioners called the need for a “more complex approach to cultural competency.” Like some US-based researchers (Kleinman & Benson, 2006; Taylor, 2003), these practitioners criticized the cultural competency model, arguing that it tended to “essentialize the relationships between culture and mental disorders” and “reproduce stereotypes” based on migrants’ nationalities. At the same time, they gradually started to advocate for an approach that mainly integrates categories of class and gender. In parallel, the Haitian experts’ participation reinforced this position during the training sessions organized by the program. Although some of them provided insights on “cultural competency” and “Haitian culture,” they emphasized in turn that “not everything can be explained by culture.” A CESFAM’s social worker revealed this skepticism in an interview:

A Haitian presenter once said that an upper-class Haitian could be very similar in his way of life to an upper-middle-class Chilean or a Frenchman living in Chile (...). We cannot think that a person gets sick in one way or another because they are Haitian or of another nationality (...). Many of the mental health problems we see here are because they are poor people or women who suffer domestic violence… not because they are Haitian or have a certain skin color.

The training sessions led practitioners to occupy three positions—not necessarily mutually exclusive—regarding the so-called “intercultural health” with migrant communities. As I mentioned earlier, many practitioners advocated against cultural competency. To a large extent, this group was characterized by more experience in the health system—at least ten years—and/or a particular affinity with Marxist-influenced social sciences and humanities. Many of them had studied at public universities. Those who were psychologists and social workers had been familiarized with traditions such as Latin American community psychology and social medicine, the psychology of liberation, the pedagogy of the oppressed, and some critical traditions within psychoanalysis and feminism. Usually, these practitioners highlighted a certain affinity between their intervention ideals and particular ways of working in public health, especially community health “on the ground” in primary healthcare (for example, interventions such as home visits, community fairs, networking with various organizations social, among others), and work with indigenous communities. In the same way, they made constant references and valued intersectional approaches to health problems.

Other practitioners instead did not advocate a reformulation of cultural competencies. They recognized the “value of culture” and promoted developing and acquiring “cultural competencies” for working with the Haitian community. At the same time, they were looking for training spaces outside the health network to learn more about Haitian-Creole medicine and Vodou and learning Creole. Generally, these practitioners tended to use culturalist understandings when analyzing the health trajectories of Haitian patients. Finally, a small group reproduced practices that adhered to mental health protocols and clinical guidelines. This led them to understand the clinical manifestations of Haitian patients within the framework of neurosciences and epigenetics prevailing in biopsychiatry in Chile (Abarca-Brown, 2023, 2024; Maino, 2022). For them, “psychiatric diagnoses” could vary in their symptomatic expression according to “culture,” but there would be “broad evidence” on the universal existence of disorders. In their opinion, “social area practitioners” should improve their “clinical eye” with Haitian patients.

## The Historical Roots of a Structural-Based Approach in Mental Health

Practitioners' skepticism and criticism of the cultural turn adopted by the Migrant Program appeared to extend beyond the ongoing debates in public health and global mental health regarding cultural and structural competency models. Their reactions did not stem from a specific engagement with current discussions on the role of structural factors in health (Hansen et al., 2018; Holmes, 2011; Kirmayer et al., 2018; Metzl & Hansen, 2014), but rather from a longstanding tradition in public health in Chile. Psychologists and social workers at the CESFAM often referred to this approach as a “way of doing mental health.” In an interview, a psychologist from CESFAM expressed the following viewpoint:

I believe that we have a community seal in public health in Chile (…). Practitioners do prevention and promotion work in the territories after their internships when they are students (...). We do home visits and mental health talks in schools and social organizations. (...). If you work in public health, you always deal with precariousness and marginality. This is not a clinic in the private sector (...). So, you see poverty and precariousness in the public institutions and patients’ lives. Sometimes you say this issue is not a mental health issue; it is a problem of poverty, of inequality of resources that affects them (…). And that is the same for everyone, for Chileans, migrants, Mapuche, etc.

Following this practitioner's words, poverty, social stratification, and socioeconomic inequalities played a crucial role in shaping patients’ afflictions and health trajectories regardless of their cultural/ethnic background. Unlike mainstream discussions on structural competency, structural violence, and structural vulnerability (Bourgois et al., 2017; Kirmayer et al., 2018; Metzl & Hansen, 2014; Stonington et al., 2018), practitioners revealed a structural approach primarily focused on class-related aspects such as poverty, social stratification, and socioeconomic inequalities. This approach tended to downplay the significance of other categories such as race, sexuality, and ability, relegating them to a secondary position. Similar findings have been observed in studies on mental health services and practices in Brazil (Ortega & Rodrigues Müller, 2022). In both Latin American cases, the predominance of class in practitioners’ structural-based approach has been yielding ground to the intersections with other categories mainly since sociopolitical events such as the murder of Marielle Franco^9^ in 2018 and the rise of Bolsonarism in Brazil during the last years (Perry, 2020), as well as the death of Joane Florvil^10^ in 2017 (Abarca-Brown, 2018) and the feminist wave in Chile in 2018 (Silva-Tapia, 2022).

The Chilean structural-based approach is characterized by the almost total omission of the category of race. Although mental health practitioners recognized the impacts of racism and violence in health—mainly based on their experiences in training modules—they tended to not integrate this category into their reflections about migrants’ afflictions and health trajectories. Practitioners used to say that the absence of these reflections responded to the “novelty” of Afro-descendant migration in Chile. For instance, according to one of the CESFAM’s social workers, associated this with a geographical aspect. He said that CESFAM’s practitioners have “little contact with indigenous population in the central part of the country (…) unlike practitioners who live in the south of Chile and work with Mapuche communities.”

Nevertheless, the omission of race has deep historical roots in the country. The birth of the Chilean nation-state at the beginning of the 19th century promoted a liberal discourse based on a blood unit or “mestizaje.” In this context, the idea of a “Chilean race” became prominent—a mixture forged between Spaniards and the indigenous people (Larraín, 2001; Subercaseaux, 2007). This new national identity valued miscegenation and, at the same time, excluded the Afro-descendant population from its national narrative, veiling racial differences from social hierarchies. Drawing on the insights of anthropologist Wade et al. (2014), it can be argued that in Chile, as in other Latin American countries, race functions as an “absent presence.” This means that while race is often erased and denied, it is present within various official spheres across Latin America. In the Chilean case, this absent presence is deeply embedded in class representations. Some researchers have described it as the “racialization of the lower classes” in Chile (Lepe-Carrión, 2017). An example of this exclusion of race is the only recent legal recognition of the “Chilean Afro-descendant tribal people” by the Chilean state in 2019.

The centrality of class aspects in practitioners’ health approach over other social categories led me to dig into its historical foundations. When I asked about professional training and the healthcare model in Chile, the same psychologist replied:

You don't apply many things you learned at university (…). But working in primary health care, you also realize that you learned a lot about social and community psychology, sometimes not at the university, but in supervisory settings, internships, study groups, and talks that you attended (...). For example, in my training, we read about Marconi^11^ and his community program in the 1960s, Paulo Freire,^12^ or about Chilean and Latin American community psychologists like Maritza Montero^13^ (...). That training helps you for working here and creating solutions to the problems that arise in precarious contexts.

This psychologist partially summarized the recent history of psychiatry and mental health and how social and health policies have shaped Otherness. Since the 1960s, the country has experienced moments marked by institutional and local community initiatives within the framework of reformist governments (1968–1973) (Araya & Leyton, 2017), including the introduction of biopsychiatry during the civil-military dictatorship (1973–1989) (Maino, 2022), and the development of mental health policies and plans with a community and territorial emphasis in a postdictatorship neoliberal context (Han, 2012). Despite these different historical, ideological and sociopolitical contexts, social and mental health policies and interventions have shaped a way of understanding Otherness preferably from the category of “class.”

Although multiple community experiences^14^ took shape in the 1960s and 1970s, Juan Marconi's Intracommunity Program (1968–1973) in southern Santiago significantly marked mental health policies and several generations of psychiatrists and psychologists in Chile (Sepúlveda et al., 2012). Marconi designed three mental health programs: “alcoholism,” “neurosis,” and “cultural deprivation” for parents and children. These programs developed community interventions in which psychiatrists, psychologists, social workers, and students worked with social organizations, community leaders, and the community.^15^ One of the most relevant interventions was Mental Health Days (“Jornadas de Salud Mental”). These were spaces where practitioners and various social actors (health practitioners, police, teachers, priests, etc.) shared knowledge on how to deal with alcoholism in the community and improve the quality of life of families (Araya & Leyton, 2017).

Marconi analyzed the Intracommunity Program in his article “The Chilean cultural revolution in mental health programs” (Marconi, 1973). Here, he used terms such as “social classes,” “capitalist society,” “social structure,” “working class,” “class struggle,” and “social change” to understand mental illness. Since his approach to mental health was marked by the category of “social structure,” the category “culture” occupied a peripheral place as a social marker in epidemiological studies (Medina & Marconi, 1970) or as a “barrier” to communication with impoverished communities (Marconi, 1969). After the 1973 coup, the Intracommunity Program was dismantled by the civic-military authorities.

After the end of the dictatorship in 1989, the state designed a mental health policy inspired by this community health tradition aiming to restructure psychiatric care based on territories and communities (Minoletti & Zaccaria, 2005). Specifically, the policy has materialized in three community-oriented mental health plans from different years: 1993, 2000, and 2017. Some researchers—some of them Marconi’s students—have pointed out that these plans and reforms can be understood as a “silent revolution” (Araya et al., 2009). That is, the implementation of a set of policies, strategies, and technologies designed to improve mental health indicators even before the call for promoting and implementing mental health programs in low- and middle-income countries (Global Mental Health Group, 2007).^16^

However, this “silent revolution” has been developed in a neoliberal context (1990s onwards). An economic rationale has framed mental health actions, delimiting clinical and community interventions to indicators such as “number of new patients registered,” “patients’ outcomes,” “health goals,” and “evidence-based therapies,” among others. Practitioners I worked with embodied these historical tensions and contradictions in their everyday practice. Some of them adopted a critical position by saying that the current mental health community approach is a “softer version” of the community psychiatry developed during the 1960s and 1970s. For instance, a psychologist interviewed provided some insights about current community interventions:

“I think we spend most of the time on individual interventions. We prioritize compliance with health goals (“metas sanitarias”).^17^ So, it's difficult to do community work as such. Community interventions are often psychoeducational interventions in the community. It is not more than that. But that is registered as ‘community intervention,’ but really that is not working 'with' the community (...). In this maelstrom, you realize that you are doing what you can. You cannot offer care with an intercultural perspective because it is difficult to stop and think about that key. That is why I believe that attention to the Chilean, the migrant, and the Mapuche does not change so much here.”

Practitioners such as this psychologist often advocated for a “community turn” during informal discussions at the CESFAM. They argued that the state should reshape mental health plans based on the traditions of community psychiatry in Chile. For these practitioners, adopting a community approach would become a means to address the “consequences” of poverty and socioeconomic inequalities in mental health. More concretely, they periodically requested the local Health Department to allocate “fewer clinical hours” and “redefine health goals.” Nevertheless, these requests were often unmet by the authorities.

Unlike mental health practitioners involved in community psychiatry experiences during the 1960s and 1970s, the current generation of practitioners, trained in the 1990s and 2000s, has increasingly embraced discussions on the interplay between class and other relevant categories, particularly gender. Rather than adopting the concept of intersectionality as an analytical tool derived from US debates in the 1990s (Collins, 2019; Crenshaw, 1989), the growing awareness among these practitioners regarding the intersections between class and gender can be attributed to the gradual introduction of a gender agenda in public policies in the country (Forstenzer, 2017; Franceschet, 2011; PNUD, 2019). Their socialization with gender aspects has gradually shaped their understanding of the complexities and interdependencies between class and gender dynamics. Since Michelle Bachelet’s first government (2006–2010), gender reforms have been implemented in areas such as the labor market, education, health, and civil rights, among others (Thomas, 2016). Thus, a gender perspective has been incorporated into all health policies and programs to guarantee gender equality and equity. For example, since 2006, gender has played a crucial role in the “Chile Crece Contigo” program, especially for women who have become mothers. In 2015, in turn, the Ministry of Health recognized gender as a social determinant of health.^18^

Introducing a gendered perspective in social and health policies seemed to have had a more significant impact on the mental health practitioners I worked with than a cultural perspective. By asking some of them who had more years of experience working in primary healthcare about the reception of a gendered perspective, they pointed out that the process was “challenging” and “useful.” Although these practitioners recalled the “resistance of some CESFAM teams”, they mentioned that the assimilation of a gendered perspective did not trigger discussions and conflicts as much as the cultural perspective. To support this position, some put forth arguments highlighting the precedence of feminism and the Chilean women's movement over the introduction of gender in health policies. They particularly underscored the women's movement's efforts in combating political violence, advocating for democracy and human rights during the 1980s (Moya-Raggio, 1984; Valenzuela, 1991).

Furthermore, while I conducted my ethnography, feminism and gender seemed to gain greater relevance in the context of the feminist strikes across the country in May 2018. The so-called Chilean feminist wave was a series of protests, demonstrations, and performances carried out mainly by high school and university students nationwide, demanding greater equality and an end to abuse and violence against women (Silva-Tapia, 2022). Many of the CESFAM’s practitioners that I worked with—especially those of the Migrant Program and the Mental Health Program—participated in these strikes and demonstrations. In this regard, Yolanda said in an interview:

"The gendered perspective is noticeable in training sessions when practitioners ask for a more complex approach to cultural competency (...). For example, mental health practitioners know that being a migrant woman is not the same as being a migrant man (...) They now have to handle situations where a female patient does not attend a medical check-up because her husband does not authorize her. Or the husband comes with her and wants to get into the midwife’s office. Things like that are very unusual in Chile (…). As it is said now, I believe that practitioners have a more intersectional approach.”

Practitioners questioned the Migrant Program's cultural turn, thus reproducing the predominance of categories such as “class” and “gender” above “culture/ethnicity/race” in social and health policies in Chile. By doing so, they redefined the initial Migrant Program goals from historical and contextual aspects (e.g., history of community psychiatry, multicultural, and gender policies) embodied both at an individual and a collective level (e.g., professional training, consequences of the dictatorship).

## Discussions and Conclusion

In this article, I have explored how the recent arrival of Afro-descendant migrants, mainly from Haiti and the Dominican Republic, has led institutions, experts, and practitioners to carry out a cultural turn in working with migrant communities in northern Santiago, Chile. This shift took form in training sessions on “cultural competency” and “Haitian culture.” I have argued that while recent local initiatives have fostered various actions to meet migrants’ health needs, health practitioners have tended to redefine cultural approaches in structural terms, mainly in terms of class—poverty, social stratification, socioeconomic inequalities—and gender. In addition, I have affirmed that this approach find its roots in a political and ideological context that provided the conditions for the development of community psychiatry experiences in the 1960s and 1970s, as well as in multicultural and gender policies promoted by the state since the 1990s.

I have tried to distance myself from public health and global mental health debates on the cultural competency and structural competency models to situate the discussion in a historical and anthropological perspective. In the past twenty years, a significant number of contributions have promoted and advocated first for the cultural competency model and then for the structural competency model, mainly in the US and Canada. However, through this ethnography, I do not intend to show a converse movement from “structure” to “culture” from the position of a particular country in the “global south.” Instead, I argue that examining how health institutions and teams conceptualize “the social” and use categories such as “culture” and “structure” enables us to appreciate the significance of local histories and contexts in contemporary Western psychiatry debates. The case of Chile, for instance, demonstrates how various institutions, experts, and practitioners engage with intersectional discussions based on their traditions within the field of public health and recent sociopolitical events (e.g., the death of Joane Florvil and the feminist wave). Recognizing this diversity becomes crucial for designing or implementing training modules in healthcare, particularly in a context where different approaches have been developed, including translational competency, intercultural competency, cultural humility, among others.

By valuing these local histories and contexts, we can carry out a decolonial exercise that questions the adoption of categories, perspectives, and models. This case highlights how health practitioners are not actors who passively adopt health models. In contrast, they assimilate, negotiate, and refuse them actively. It also encourages institutions and practitioners to promote “good practices” from their own local histories and contexts—community psychiatry, particularly. This perspective allows us to examine how the adoption of specific competency models not only reveals issues related to the relationship between institutions, health teams, and communities but also the politics of recognition involved (Giordano, 2014) and forms of multiculturalism in specific contexts. Finally, by highlighting local histories and context, we can distance ourselves from an understanding of social categories—for example, culture, race, class, and gender—as static or self-evident entities (Adams et al. 2019; Briehl, 2003; Yates-Doerr, 2018) to understand them as mutable, hybrid, synergistic, and variable categories.

## Footnotes

1 I am referring to the governments of Eduardo Frei Montalva (1964–1970) and Salvador Allende Gossens (1970–1973).

2 There are an extensively literature on cultural competency and structural competency models. For cultural competency, see: Betancourt et al. (2016), Cross et al. (1989), Jeffreys (2015), Kirmayer (2012a, 2012b), Mareno and Hart (2014); and for structural competency, see: Hansen et al. (2018), Kirmayer et al. (2018), Martínez-Hernáez and Bekele (2022), Metzl et al. (2017), Metzl and Hansen (2018).

3 This research was approved by the Ethics Committee of King’s College London (Ethics Ref: HR-17/18-5319) and was authorized by the local Health Department.

4 Yolanda and other names in this work are pseudonyms.

5 With this budget, the program also created informative material to improve migrants’ access to health services.

6 Mapuche language.

7 It is estimated that approximately 50% of the population has access to healthcare in Haiti. For some researchers, the low level of access to health services would partly explain the legitimacy of Haitian-Creole medicine and Vodou in Haiti.

8 The “Chile Crece Contigo” program gives children access to health and social services and benefits in different state levels from gestation to age nine. The program’s premise is that children build the foundations of learning, language, physical health, mental health, and socioemotional development during their first year of life.

9 Marielle Franco (1979–2018) was a Brazilian black left-wing politician. She was killed by two murdered traveling in another vehicle.

10 Joane Florvil (1989–2017) was a Haitian woman who died under strange circumstances while in custody at a police station in Santiago, Chile.

11 Juan Marconi (1924–2005) was a Chilean psychiatrist and epidemiologist. He developed the Intracommunity Program (1968–1973) in the south of Santiago.

12 Paulo Freire (1921–1997) was a Brazilian educator and philosopher. He wrote the book “Pedagogy of the Oppressed” (1968) during his stay in Chile.

13 Maritza Montero is a Venezuelan social psychologist. Her contributions focus on community psychology, critical psychology, and Liberation Psychology in Latin America.

14 I am referring to Martin Cordero’s community experience and his team in Temuco, as well as Luis Weinstein’s experience called “Salud Mental Poblacional” in Santiago.

15 Marconi organized the community work based on the “role delegation pyramid” (“pirámide de delegación de funciones”). The pyramid assigned roles and tasks to the entire community in general and responsibilities to community leaders to work with alcoholism (Marconi, 1971).

16 For example, their second plan promoted a series of strategies to increase access to mental healthcare in the population. In 2001, the Ministry of Health implemented the National Program for the Detection, Diagnosis, and Treatment of Depression in primary healthcare. This program was the first in a lower-middle-income country (Araya et al., 2009). In 2004, the State promulgated the Explicit Health Guarantees Law (GES, in Spanish) to strengthen the social protection system. This law guarantees access, opportunity, financial protection, and quality of care for 80 health conditions. Among them are schizophrenia, depression, bipolar disorder, and substance addiction.

17 The law 19.813 established an evaluation system called “metas sanitarias” (“health goals”) through which primary healthcare centers are evaluated annually. Based on the collective performance of the centers, monetary incentives are established for the officials.

18 Exempt Resolution N°136 of March 30, 2015.
